# Prognostic significance of p53 immunohistochemical expression in adenoid cystic carcinoma of the salivary glands: a meta-analysis

**DOI:** 10.18632/oncotarget.15297

**Published:** 2017-02-11

**Authors:** Qinglin Li, Ping Huang, Chuanming Zheng, Jiafeng Wang, Minghua Ge

**Affiliations:** ^1^ Department of Pharmacy, Zhejiang Cancer Hospital, East Banshan Road, Hangzhou, Zhejiang Province, China; ^2^ Department of Head and Neck Surgery, Zhejiang Cancer Hospital, Hangzhou, Zhejiang Province, China; ^3^ Zhejiang Key Laboratory of Head and Neck Tumor, Zhejiang Cancer Hospital, Hangzhou, Zhejiang Province, China

**Keywords:** adenoid cystic carcinoma, salivary glands, p53 expression, prognosis, meta-analysis

## Abstract

Adenoid cystic carcinoma of salivary glands is a rare adenocarcinoma and has been placed in “high-risk” category as poor long-term prognosis. The purpose of this study was to investigate p53 protein expression in adenoid cystic carcinoma of salivary glands and its correlation with clinicopathological parameters and prognosis. Literatures were searched from PubMed, Embase, Cochrane Library and Web of Science, which investigated the relationships between p53 expression and pathological type, clinical stage, local recurrence, metastasis, nerve infiltration and overall survival. A total of 1,608 patients from 36 studies were included in the analysis. The results showed that p53-postive expression rate was 49% in adenoid cystic carcinoma of salivary glands (OR=10.34, 95%CI: 4.93-21.71, *P* < 0.0001). The p53-postive expression was closely related to tumor types (OR=0.30, 95%CI: 0.14-0.65, *P* < 0.0001). The tumor with solid histological subtype had a strong positive correlation with p53 expression. The combined analysis revealed that the p53-positive expression rate among patients in T1and T2 stage was 41.4%, compared to 53.2% among those in T3 and T4 stage. However, there was no significant correlation between tumor stage and p53 expression (OR=0.47, 95% CI: 0.17-1.29, *P* = 0.14). Besides, compared to patients with p53-negative expression, those with p53-positive expression had a greater chance of developing metastasis, local recurrence and nerve infiltration as well as poorer 5-year overall survival (*P* < 0.01). In conclusion, the p53 expression is related to the survival of adenoid cystic carcinoma of salivary glands. It can be considered as the auxiliary detection index in treatment and prognosis of adenoid cystic carcinoma of salivary glands.

## INTRODUCTION

There is significant difference in the incidence of salivary gland tumors in different countries, which is 0.15-1.6/100 thousand in China according to the WHO report. Malignant salivary gland tumors constitute less than 1% of all the malignancies while 21-46% of all salivary gland tumors [1, 2]. Adenoid cystic carcinoma (ACC) is a rare variant of adenocarcinoma that most often arises in the salivary glands [3], and comprises approximately 1 % of all malignant tumors of oral and maxillofacial origin [4]. Adenoid cystic carcinoma (ACC) constitutes approximately 4% of salivary epithelial tumors, which is the most common malignant tumor of minor salivary glands and the second most common malignant tumor involving major salivary glands. Almost 70% of minor salivary gland tumors are malignant, and ACC is the most common type [5, 6]. It can occur at any age, but 40-60 years of age are most commonly affected, and there are slightly more women than men. The three major growth patterns of salivary glands adenoid cystic carcinoma (SACC) are tubular, cribriform, and solid. One of the important prognostic factors is the histological grade, which is determined by the solid tumor component percentage [7]. The clinical and pathological characteristics include slow growth, perineural invasion, distant metastasis, and potential local recurrence [8, 9]. The natural history of SACC is best described that 10-year overall survival (OS) for patients is about 50%, but locoregional and distant recurrence after a short disease-free interval are common [9, 10]. Once metastatic disease is present, case series of salivary ACC suggest that the median duration of survival is about 3 years. Although complete surgical resection and additional radiotherapy have been shown to improve long-term survival, the prognosis of adenoid cystic carcinoma remains poor [11, 12]. Accordingly, it is very important to objectively predict prognosis of patients with tumor resection and the development of metastasis. To improve patient outcomes, it is important to identify clinical markers that may predict prognosis and response to specific therapies. However, there are no established biomarkers that correlate with the outcome and therapeutic response in patients with SACC [13].

Because of the rarity of SACC, there are few clinical trials investigating the prognostic indicator. The aim of this systematic review was to assess the association between p53 immunohistochemical expression and pathological types, clinical stage, local recurrence, metastasis, nerve infiltration and survival, to observe the expression of p53 in SACC and its correlation with clinicopathological parameters and prognosis, which may be useful for planning the management and assessing the prognosis.

## RESULTS

### Study selection procedures

**Figure 1:**
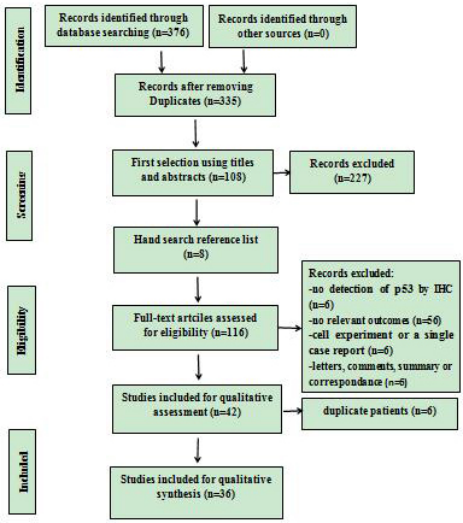
Flowchart of the study selection

The study selection procedure is presented in Figure 1. In the initial literature search, 376 studies matched the search terms, of which 41 studies were excluded due to overlapping data sets. And 227 studies were ruled out because apparent irrelevance was found when reading the title and/or abstract. Additional 8 relevant studies from the reference list were included. By reading through the full text of the remaining studies, 74 studies were excluded ( p53 was not detected by IHC in 6 studies, 56 studies had no relevant outcomes, 6 studies were cell experiments or a single case report, and 6 studies were letters, summary, comments or correspondences). Moreover, 6 studies were ruled out due to duplicate patients. Finally, there were 36 articles left with sufficient data for extraction.

### Study characteristics

Thirty-six studies [2, 6–7, 12, 14–45] published between 1996 and 2015 were eligible for this meta-analysis. The total number of patients included was 1,608, ranging from 10 to 219 patients per study. The major characteristics of the 36 eligible publications are reported in Table 1. The studies were conducted in 13 countries and 5 continents (Asia: China, Japan, Korea, Pakista, Israel, Iraq; Europe: Finland, Noraway, Sweden, Italy, Germany; North America: USA; South America: Brazil). p53 expression was detected in all patients in the eligible studies using IHC. The control group was set up in 23 studies, including tissues of normal salivary glands, benign tumour of salivary glands or paracancer tissues. Tumor stage was evaluated in 20 studies, and histopathological types of SACC were provided in 23 studies.

**Table 1:** Main characteristics of all the studies included in the meta-analysis

First Author, Year	Origin County	Control Source	Detection Method	Tumor Stage	No. of patients	Number of samples			Histopathological pattern			
						SACC	Control	Cribriform	Tubular	Cribriform and Tubular	Solid	
Guo S, 2002	China	NSG	SP	I-IV	57	45	12	23	17	40	5	8
Peng X, 1998	China	NSG	SP	I-IV	60	50	10	24	22	46	4	8
Danyel, 2006	Brazil	NS	SP	I-IV	107	107	NS	58	27	80	22	6
Kazuaki C, 2007	Japan	NS	SP	I-IV	27	27	NS	12	8	20	7	6
Gabriele, 2005	Italy	NSG	SP	I-IV	42	21	21	11	2	13	8	8
Liu F, 2004	China	BSG	SP	NS	52	47	5	17	12	29	18	8
Lin J, 2004	Japan	NS	SP	NS	39	39	NS	17	13	30	9	8
Jose E, 2008	USA	NS	SP	NS	47	47	NS	36	4	40	7	6
He W, 2012	China	BSG	SP	I-IV	46	34	12	NS	NS	14	20	7
Vesa J.k, 1997	Finland	BSG	SP	I-IV	219	103	116	NS	NS	NS	NS	7
Helen P, 1996	USA	NS	NS	NS	13	13	NS	NS	NS	NS	NS	5
Wajiha A, 2011	Pakista	NS	SP	I-III	40	40	NS	18	1	19	5	6
Zhang DS, 2005	China	NSG	SP	I-IV	40	38	2	NS	NS	32	6	7
Rieko D, 1999	Japan	NS	SP	NS	31	31	NS	11	17	28	3	6
T.Kiyoshima, 2001	Japan	NS	SP	NS	17	17	NS	3	8	11	6	6
O Ben-lzhak, 2007	Israel	Paracancer	SP	NS	66	66	66	NS	NS	NS	NS	6
Wang XF, 2014	China	Paracancer	SP	I-IV	36	36	10	15	9	24	1	8
Wei WT, 2007	China	NS	SP	I-IV	39	39	NS	NS	NS	NS	NS	5
Natheer H, 2009	Iraq	BSG	SP	I-IV	12	12	10	3	6	9	3	8
YUZOY, 1996	Japan	NS	NS	I-IV	21	21	NS	7	2	9	12	6
Zhu YM, 2003	China	NS	SP	NS	36	36	NS	19	13	32	4	6
Marina, 2009	Brazil	NSG	SP	II-IV	26	22	4	13	2	15	7	8
Wang YS, 2011	China	BSG	SP	NS	45	35	10	NS	NS	NS	NS	6
Hu HQ, 2013	China	NSG.	SP	NS	32	16	16	NS	NS	NS	NS	6
F. A. A, 2004	Brazil	BSG	SP	NS	30	15	15	NS	NS	NS	NS	6
Kyoichi K,2013	Japan	NS	SP	I-IV	32	32	NS	NS	NS	23	7	5
Q. R. Z, 1997	China	NS	SP	I-IV	27	27	NS	NS	NS	NS	NS	5
Stalen N, 1997	Norway	Paracancer	SP	I-IV	41	41	41	NS	NS	NS	NS	7
ANDERS N, 2000	Sweden	BSG	SP	NS	123	55	68	NS	NS	NS	NS	6
Liu H, 2013	China	NSG	SP	NS	50	40	10	NS	NS	NS	NS	6
MWengh- oefer,2008	Germany	NSG	SP	NS	10	3	7	NS	NS	NS	NS	7
Min J, 2013	Korea	BSG	SP	NS	20	10	10	NS	NS	NS	NS	6
L.C.J, 2014	China	NS	SP	I-IV	35	35	NS	NS	NS	NS	NS	5
Chen J G, 1999	China	NSG	SP	IV	40	20	20	NS	NS	8	12	6
Carolina C, 2012	Brazil	BSG	SP	NS	18	4	14	NS	NS	NS	NS	6
MaoMing, 2002	China	NSG	SP	NS	32	22	12	NS	NS	NS	NS	5

Abbreviations: SACC: salivary glands adenoid cystic carcinoma, NSG: normal salivary glands, BSG: benign tumors of salivary glands, NS: not stated, SP: streptavidin-perosidase in imuunohistcochemical staining.

### Meta-analysis

#### Meta-analysis of p53 expression between the SACC group and the control group

**Figure 2:**
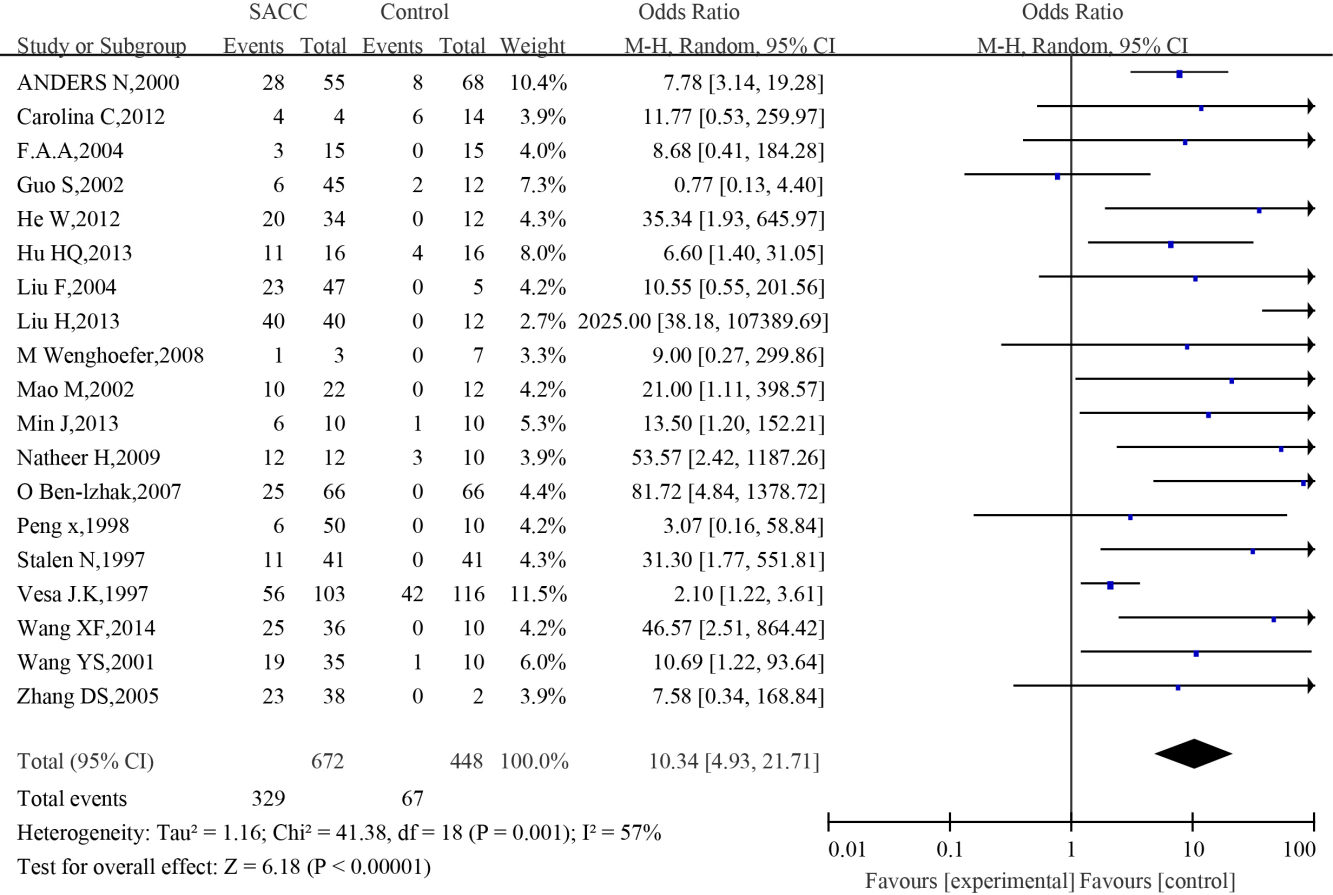
The forest plot of the meta-analysis of p53 expression between the adenoid cystic carcinoma of the salivary glands group and the control group

p53 positive expression in the adenoid cystic carcinoma of the salivary glands group and the control group (normal salivary glands, benign tumor of salivary glands or paracancer tissues) were compared in nineteen eligible studies, with 672 cases of SACC tissues and 329 cases of control tissues. It was found that p53 was positive in 448 cases and 67 cases, respectively. The heterogeneity of the 19 studies was tested, and the statistically significant heterogeneity was observed between the studies (I^2^=57%, *P*=0.001). The results showed that p53 was expressed at a high level in SACC tissues (OR=10.34, 95%CI: 4.93-21.71, *P*<0.00001)(Figure 2).

**Figure 3:**
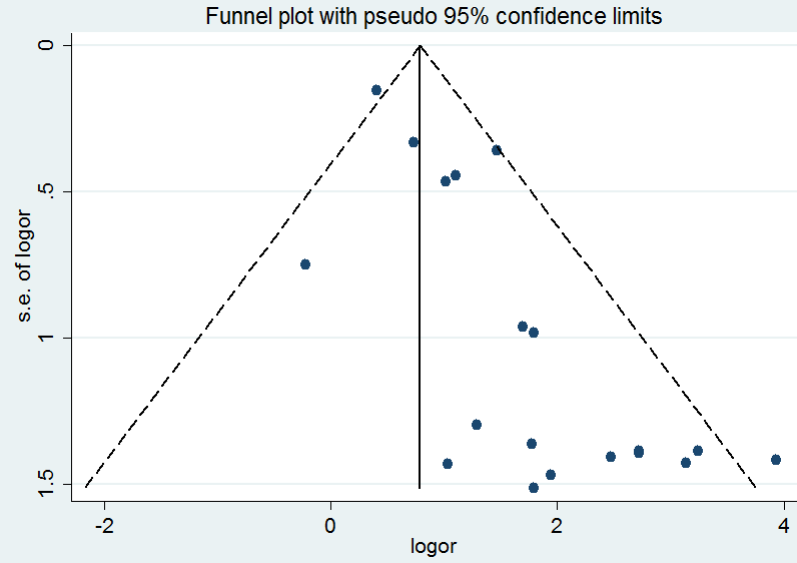
Begg's funnel plot of the potential publication bias of included studies

Sensitivity analysis did not show any significant difference when meta-analysis was repeated after each individual study was omitted or after the studies were excluded. Begg's funnel plot was performed to assess the publication bias in the literature. All 19 eligible studies investigating p53 expression between the SACC group and the control group yielded a Begg's tests core of P=0.401, according to the funnel plot (Figure 3), and there was no publication bias in these analyses.

#### Meta-analysis of p53 expression in the different histopathological types of SACC

**Figure 4:**
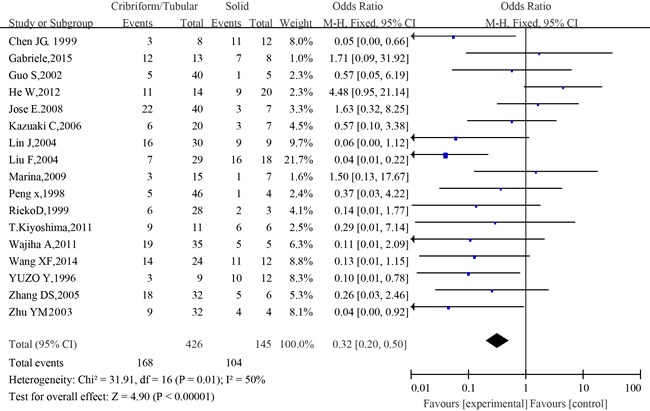
The forest plot of the meta-analysis between p53 expression in the cribriform/ tubular and solid types of salivary glands adenoid cystic carcinoma

17 of 36 studies investigated the association between p53 expression and histopathological types of patients with adenoid cystic carcinoma of the salivary glands. The combined analysis showed that the positive expression rate of p53 in cribriform/ tubular SACC was 39.4% (168/426 cases), and that in solid SACC was 71.7% (104/145). Statistically significant heterogeneity was observed between the studies (I^2^=50%, *P*=0.01). The combined analysis showed that the patients of the cribriform/ tubular type had a low expression of p53, and those of the solid type had a high p53 expression(OR= 0.32, 95 % CI: 0.20 −0.50, *P*<0.00001)(Figure 4).

**Figure 5:**
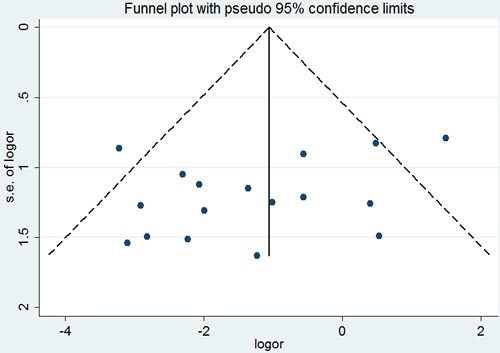
Begg's funnel plot of the potential publication bias of included studies

Sensitivity analysis did not show any significant difference when meta-analysis was repeated after each individual study was omitted or after excluding studies. Regarding the publication bias in the studies, no funnel plot asymmetry was found. Furthermore, Begg's test was applied to provide statistical evidence for funnel plot symmetry. As expected, the P value of Begg's test was 0.303 (Figure 5). Hence, there was no evidence for significant publication bias in the meta-analysis.

#### Meta-analysis of p53 expression in the different tumor stages of SACC

**Figure 6:**
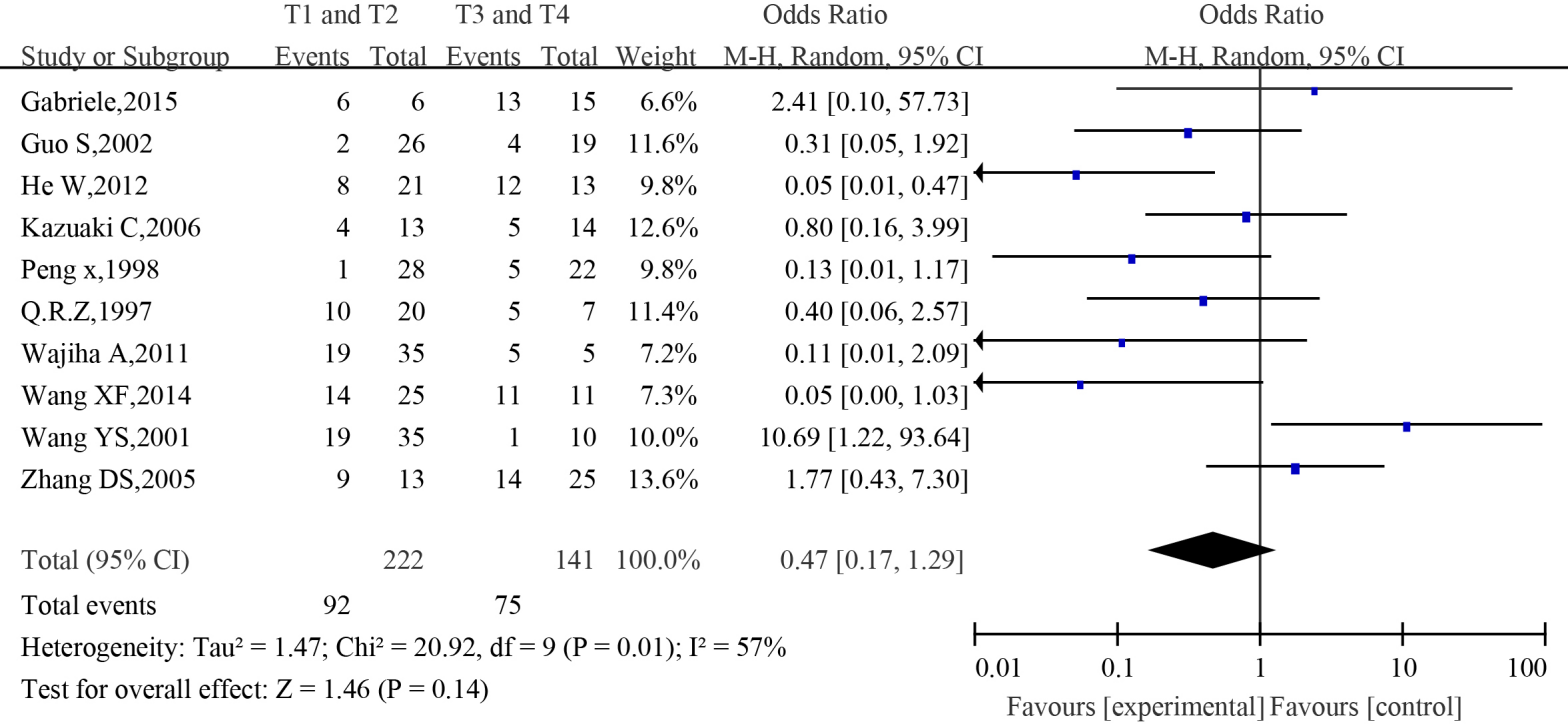
The forest plot of the meta-analysis between p53 expression in the T1/T2 and T3/T4 of salivary glands adenoid cystic carcinoma

The association between p53 positive expression and tumor stage of SACC patients was investigated in 10 of 36 studies. The combined analysis showed that the positive expression rate of p53 among patients in T1and T2 stage was 41.4% (92/222 cases), and that among patients in T3 and T4 stage was 53.2% (75/141 cases). The heterogeneity of the 10 studies was tested, and the statistically significant heterogeneity was observed between the studies (I^2^=57%, *P*=0.01). The combined analysis showed that the positive expression rate of p53 in tumor in T3 and T4 stage was higher than that in T1 and T2 stage, however, no statistically significant correlation was observed between tumor stage and p53 expression (OR=0.47, 95%CI:0.17-1.29, P=0.14)(Figure 6).

**Figure 7:**
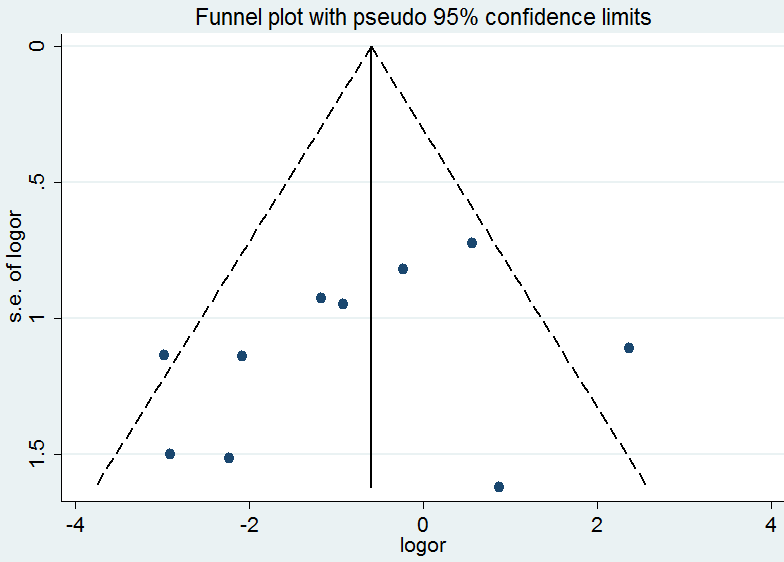
Begg's funnel plot of the potential publication bias of included studies

No significant difference was observed among the studies in sensitivity analysis. At the same time, no funnel plot asymmetry was found in the studies and the Begg's test did not show any evidence of publication bias (*P*= 0.371; Figure 7)

#### Meta-analysis between p53 expression and metastasis of SACC

**Figure 8:**
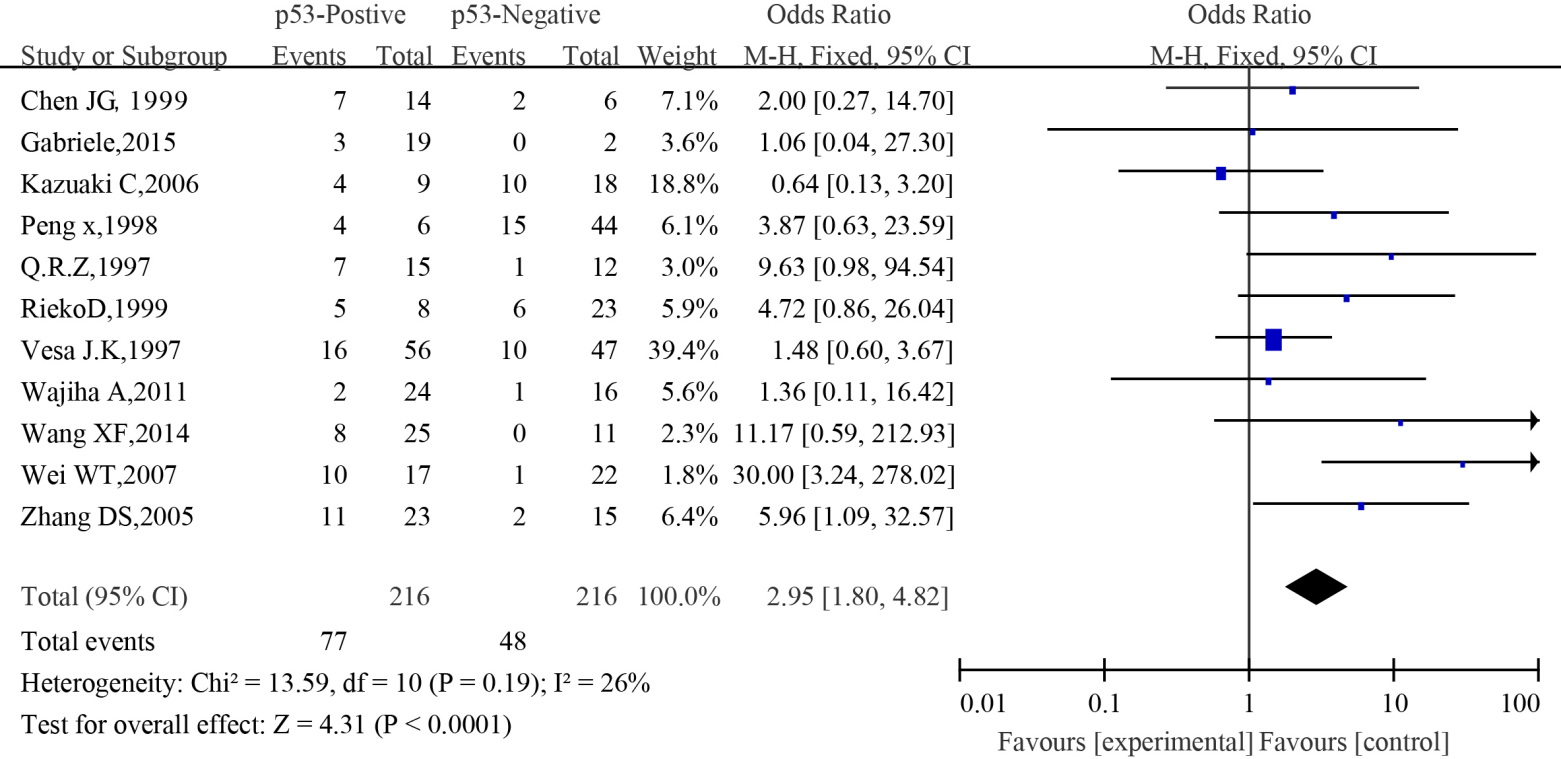
The forest plot of the meta-analysis between p53 expression and metastasis of salivary glands adenoid cystic carcinoma

The information concerning the association between p53 expression and metastasis of adenoid cystic carcinoma was provided in eleven studies. The combined analysis showed that the metastatic rate of patients with positive expression of p53 was 35.6% (77/216), and that of patients with negative expression of p53 was 22.2% (48/216). No significant heterogeneity was observed among the studies on p53 expression and metastasis (I^2^=26%, *P*=0.19). The combined analysis showed that compared to patients with negative expression of p53, those with positive expression of p53 had a greater chance of developing metastasis (OR=2.95, 95%CI, 1.80–4.82, *P*<0.0001) (Figure 8).

**Figure 9:**
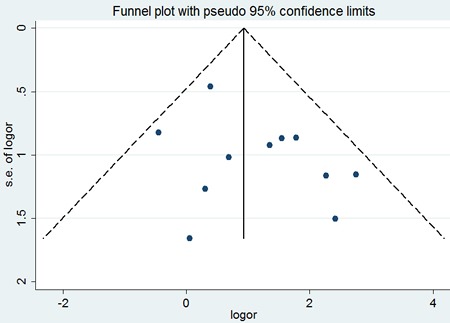
Begg's funnel plot of the potential publication bias of included studies

Similarly, no significant difference was observed among the studies in sensitivity analysis (Figure 12). At the same time, no funnel plot asymmetry was found in the studies and the Begg's test did not show any evidence of publication bias (*P*= 0.755; Figure 9).

#### Meta-analysis between p53 expression and local recurrence of SACC

**Figure 10:**
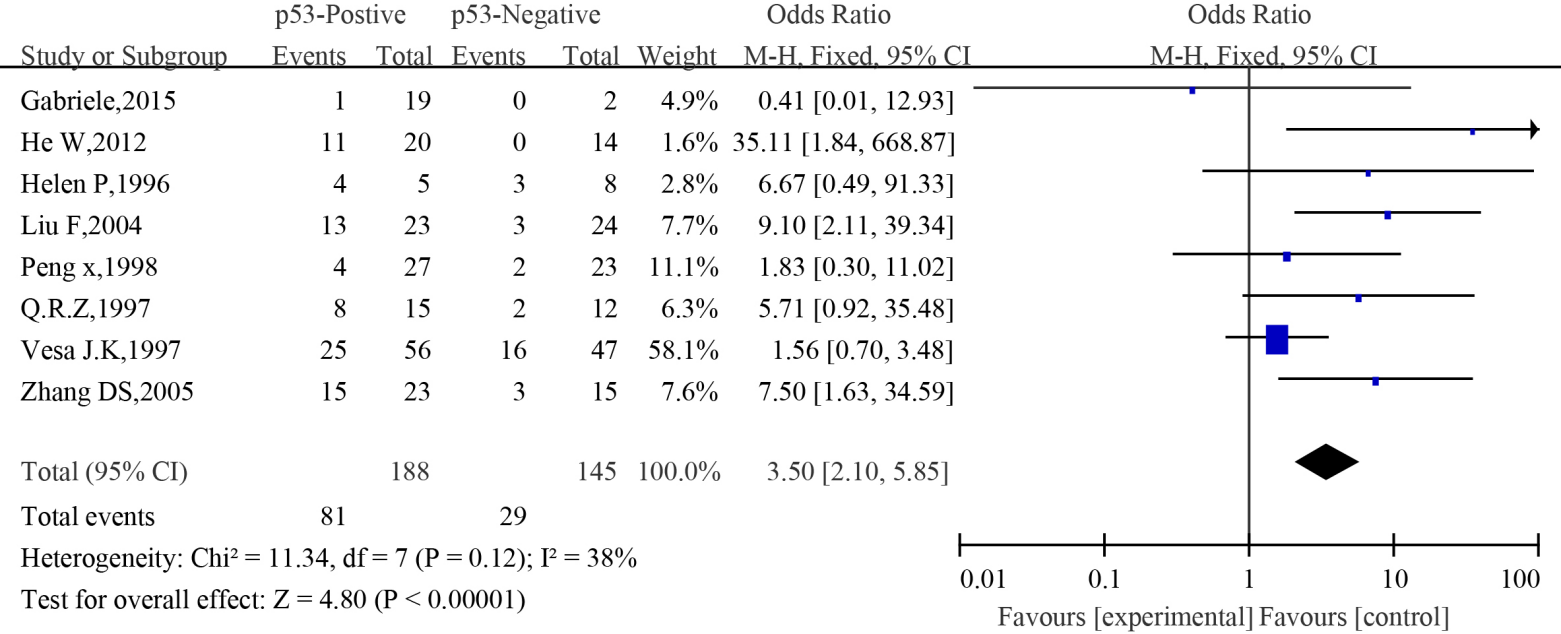
The forest plot of the meta-analysis between p53 expression and local recurrence of salivary glands adenoid cystic carcinoma

Eight eligible studies provided information concerning the association between p53 expression and local recurrence of SACC. The combined analysis showed that recurrence rate of patients with p53 positive expression was 43.1%(81/188), and patients with p53 negative expression was 20%(29/145). A slight heterogeneity was observed in the pooled studies (I^2^=38%, *P*=0.12). The combined analysis showed that compared to patients with negative expression of p53, those with positive expression of p53 had a significantly higher incidence of local recurrence (OR=3.50; 95 % CI:2.10-5.85, *P*<0.00001) (Figure 10).

**Figure 11:**
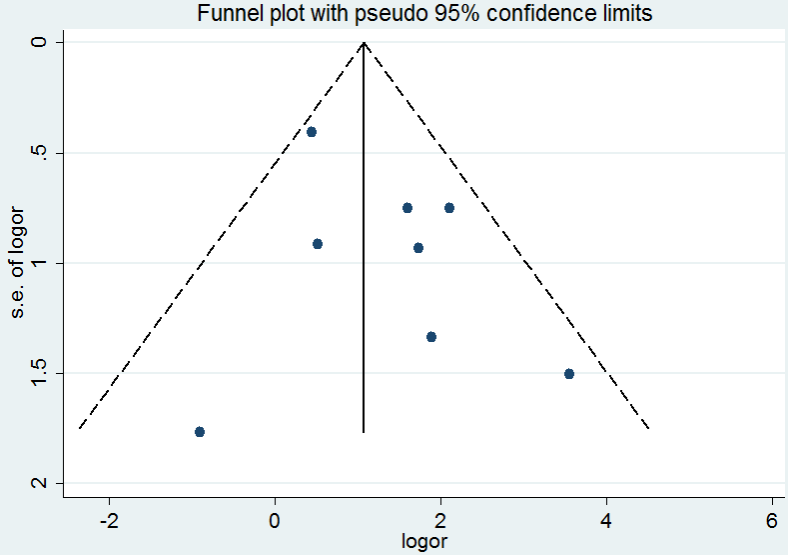
Begg's funnel plot of the potential publication bias of included studies

Similarly, no significant difference was observed among the studies in sensitivity analysis. Similar results were found for investigating association between p53 positive expression and recurrence (a Begg's test score of *P*=1.000). Meanwhile, according to the funnel plot (Figure 11), there was no publication bias in these analyses.

**Figure 12:**
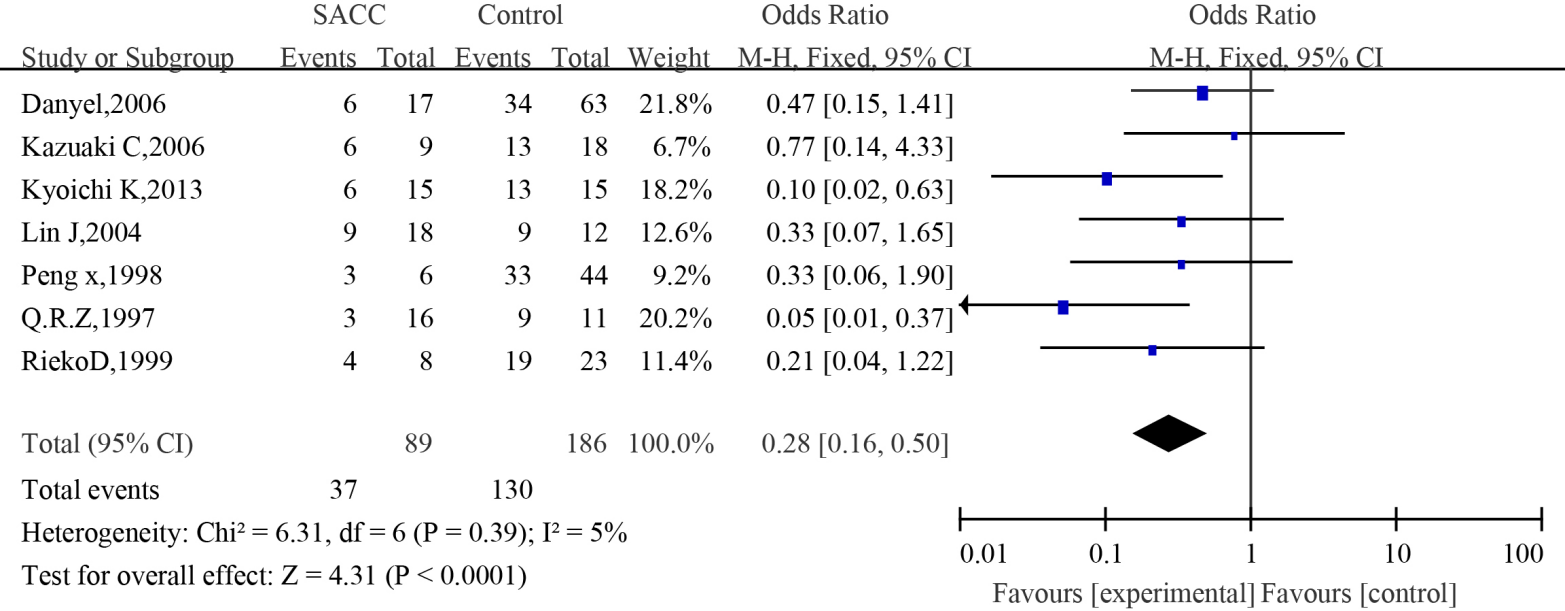
The forest plot of the meta-analysis between p53 expression and 5-year survival of salivary glands adenoid cystic carcinoma

#### Meta-analysis between p53 expression and 5-year survival of SACC

The information concerning the association between p53 expression and 5-year survival of SACC was revealed by seven eligible studies. The combined analysis showed that the 5-year survival rate of patients with positive expression of p53 was 41.76% (37/89) compared to 70% (130/186) of patients with negative expression of p53. A slight heterogeneity was observed in the pooled studies (I^2^=5%, P=0.39). Meta-analysis results showed that compared to patients with negative expression of p53, those who had positive expression of p53 had significantly poorer OS (OR=0.28; 95% CI, 0.16–0.50, P<0.0001)(Figure 12).

**Figure 13:**
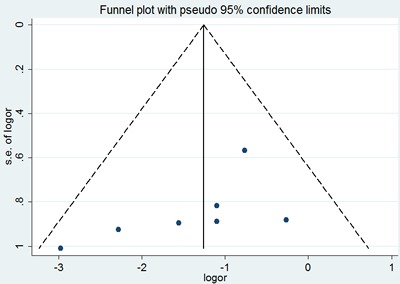
Begg's funnel plot of the potential publication bias of included studies

Similarly, no significant difference was observed among the studies in sensitivity analysis. Begg's test was not significant, and the funnel plot for 5-year survival showed only slight asymmetry, which reduced the likelihood of significant publication bias(Figure 13).

#### Meta-analysis between p53 expression and nerve infiltration of SACC

**Figure 14:**
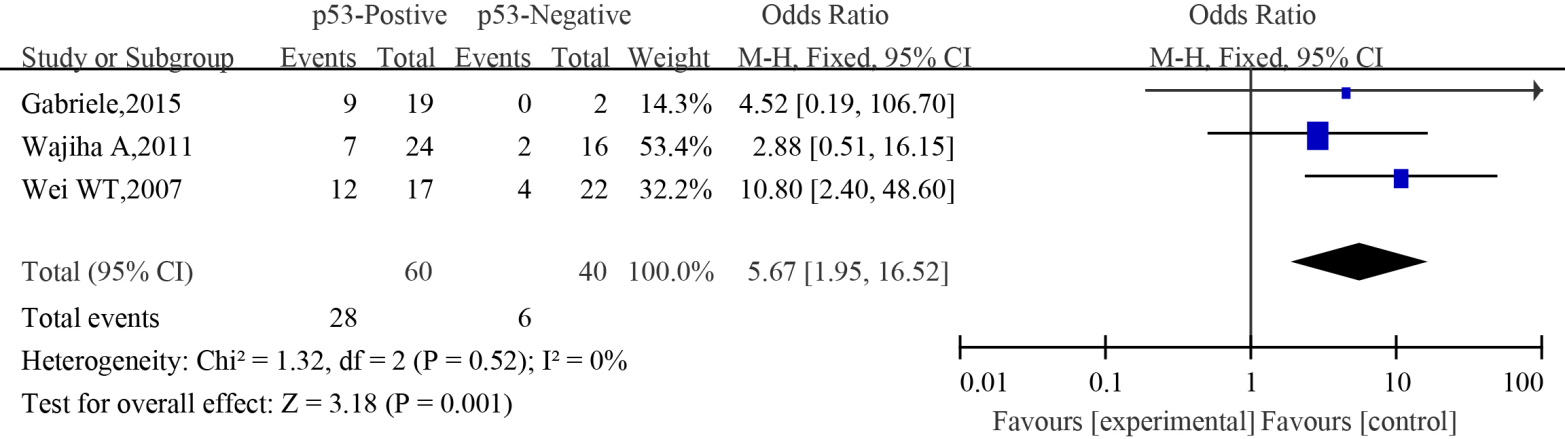
The forest plot of the meta-analysis between p53 expression and nerve infiltration of salivary glands adenoid cystic carcinoma

Three eligible studies provided information concerning the association between p53 expression and nerve infiltration of SACC. The combined analysis showed that the nerve infiltration rate of patients with positive expression of p53 was 46.7% (28/60), and that of patients with negative expression of p53 was 15% (6/40). No significant heterogeneity was observed among the studies on association between p53 expression and nerve infiltration (I^2^=0, *P*=0.725). The combined analysis showed that compared to patients with negative expression of p53, those with positive expression of p53 had more chance of developing nerve infiltration (OR=3.03; 95%CI, 1.42–6.43, *P*=0.002)(Figure 14).

**Figure 15:**
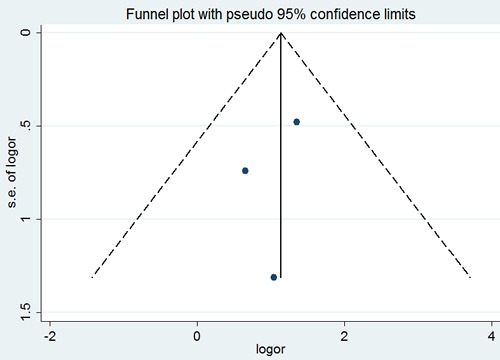
Begg's funnel plot of the potential publication bias of included studies

Similarly, no significant difference was observed among the studies in sensitivity analysis. Similar results were found for investigating association between p53 positive expression and nerve infiltration (a Begg's test score of *P*=1.000). Meanwhile according to the funnel plot (Figure 15), there were no publication bias in these analyses.

## DISCUSSION

### Principal findings

In this study, the relationship between p53 immunohistochemical expression and pathological types, clinical stage, local recurrence, metastasis(lymph node metastasis and distant metastasis), nerve infiltration and survival was statistically analyzed in order to investigate the association between positive expression of p53 and prognosis of SACC. The present meta-analysis has combined 36 publications including 1,608 patients to yield statistics, showing the following results: p53 was expressed at a high level in SACC tissues and the positive expression rate was 49% (OR= 10.34, 95%CI:4.93-21.71, *P*<0.00001). The p53-positive expression was closely related to tumor types (OR=0.32, 95 % CI:0.20-0.50, P<0.00001). Those with solid histological subtype tumor had a strong positive correlation with p53 expression. The combined analysis showed that positive expression rate of p53 among patients in T1and T2 was 41.4%, and that among patients in T3 and T4 stage was 53.2%. However, no statistically significant correlation was established between tumor stage and p53 expression (OR=0.47, 95%CI: 0.17-1.29, *P*=0.14). Besides, when compared to patients with negative expression of p53, those with positive expression of p53 had more chance of developing metastasis, local recurrence and nerve infiltration as well as poorer 5-year OS (*P*<0.01).

### Overview of the evidence

In the present meta-analysis, most of the ORs from the included studies were from multivariate analyses adjusting for confounding factors. Indeed, the adjusted ORs are more accurate than the unadjusted ORs since they reduce the risk of bias from other possible confounding factors. Hence, the findings from the present meta-analysis provided strong evidence that could establish a correlation betweeen p53 protein expression and clinicopathological parameters in prognostic assessment of SACC at microscopic level. Finally, of note, our included study population was mainly from four continents including thirteen countries. Therefore, the present study extensively revealed the value of p53 protein expression in the prognosis of patients with SACC.

### Outcome and Prognosis

In our study, the p53 positive expression rate of SACC was higher than that in control tissues (P=0.001), which indicated that p53 plays an important role in the development of SACC. In the solid type, the positive expression rate of p53 was higher than that of the cribriform and tubular type (*P*<0.01). However, there was no significant difference between the cribriform and tubular type (*P*>0.05). The prognostic effects of solid histopathology are controversial. Some believes it means a lower rate of survival when compared with the tubular or cribriform type, whereas others have shown that there is no relationship [46, 47]. The combined analysis showed that the recurrence rate of patients with positive expression of p53 was 43.1%, and that of patients with negative expression of p53 was 20%. Compared to patients with negative expression of p53, those with positive expression had more chance of developing local recurrence (*P*<0.01), indicating that the expression of p53 was closely related to the recurrence, which may be an important marker to reflect the biological characteristics of ACC and to judge the prognosis. Similarly, Helen et al demonstrated that the expression of p53 was closely related to the degree of differentiation and recurrence of ACC [24]. Through this study, it was concluded that the expression of p53 in SACC was related to the degree of differentiation and malignancy, p53 positive rates, poor differentiation, high degree of malignancy and poor prognosis.

Many studies reported presence of metastasis in SACC but no study has been conducted to demonstrate any correlation between metastasis (lymph node metastasis and distant metastasis) and p53 expression so far. In our study, it was found that compared to patients with negative expression of p53, those with positive expression of p53 had more chance of developing metastasis, and 35.6% of the cases with positive expression of p53 exhibited metastasis. There was significant correlation between p53 expression and metastasis (p=0.001). It was suggested that overexpression of p53 protein was a high risk marker for the distant metastasis of salivary glands.

The combined analysis revealed that the positive expression rate of p53 among patients in T1and T2 stage was 41.4%, and that among patients in T3 and T4 stage was 53.2%. However, overall no statistically significant correlation was established between tumor stage and p53 expression (*p*=0.14). Similarly, Coggi G et al. demonstrated that the overexpression rate of p53 in T4 and T3 tumor was higher than that in T1 and T2 tumor. Therefore, they believed that the overexpression of p53 was the characteristic of advanced cancer [48]. Conversely, Gallo O et al. concluded that p53 overexpression and clinical stage of SACC were not related to each other [49]. Therefore, we think that the abnormal expression of p53 protein may not be an indicator of clinical stage.

The present meta-analysis combined seven publications including 275 patients to yield statistics, and indicated a significant role of p53 detected by IHC in predicting 5-year overall survival of SACC. The study showed that the 5-year survival rate of patients with positive expression of p53 was lower than that of patients with negative expression of p53(*P*<0.001), which may be related to the sensitivity of tumor cells to radiotherapy and chemotherapy besides the predisposing invasiveness, local recurrence and metastasis of tumor cells with overexpression of p53.

### The potential tumorigenesis mechanism and prognostic value of p53-postive expression

Tumor suppressor gene *p53* is one of the most tumor relevant genes to date, which is the most frequently mutated gene in human tumor [50]. In normal cells, *p53* gene plays important physiological roles through encoding wild-type p53 protein. Wild-type p53 protein combines with multiple cancer proteins, inhibiting their effects on cell transformation. As a transcription factor, wild-type p53 protein can regulate expression of multiple genes, such as enhancing *p21* gene expression and inhibiting *bcl-2* gene expression, and then inhibit cellular division and proliferation. Wild-type p53 protein can induce cell apoptosis through enhancing *Bax* gene expression and inhibiting *bcl-2* gene expression, which can promote DNA damage repair through activating Gadd to sustain genome DNA integrity. Mutant p53 protein not only loses the primitive function of tumor suppression, but also may gain the function of oncogenes, such as causing over-proliferation and division of cells, leading to cellular malignant transformation, tumor development, increased tumor invasion, as well as radiotherapy and chemotherapy resistance [51]. The half-life of wild-type p53 protein is only 6min-12min, and its content is very low in nuclei, which can be hardly detected by regular immunohistochemistry (IHC) method. However, the half-life of mutant-type p53 protein is as long as 4h-12h, and it is accumulated in nuclei with high content. In other words, p53 protein is overexpressed, which is also the main product detected by IHC.

In our study, the positive rate of p53 protein overexpression was relatively high among various pathological types of SACC. It has been found that the overexpression of p53 gene is involved in selective tolerance of tumor cells to chemotherapeutic drugs. The multidrug resistance associated gene of tumor cells is mainly mdrl, and the wild-type p53 protein can inhibit the promoter activity of mdrl gene, and the mutant p53 gene protein can enhance the promoter activity. In order to obtain a good therapeutic effect, mdrl unrelated drugs, such as paclitaxel, may be used for the malignant tumor with overexpression of p53 [52]. In addition, overexpression of p53 can induce tumor cells to have radiation tolerance, thus the tumor is not sensitive to radiotherapy [53].

### Limitations

Study selection and data extraction were performed independently and reproducibly by two reviewers. We also explored heterogeneity and potential publication bias in accordance with published guidelines. The present meta-analysis may have several limitations that need to be addressed: Firstly, in spite of the comprehensive search strategy, we can not avoid the possibility of having missed relevant studies, in particular studies published in languages other than English and Chinese. Positive results tend to be accepted by journals, while negative results are often rejected or not even submitted. There may have been negative studies that were never published, and the original data of several studies could not be obtained. Secondly, although this study has tried to collect all the relevant data, the potential publication bias is inevitable, and some data may be missed. The missed information may reduce the reliability of p53 expression as a prognostic indicator of SACC. Thirdly, the p53 positive standards of the included studies were not uniform, and some studies did not even give the specific criteria, which may also cause the heterogeneity of the studies. Fourthly, our analyses were based on retrospective studies rather than prospective studies, thus it was hard to effectively avoid recall and selection bias. Finally, our meta-analysis relied on publication other than individual patient data. Studies may have differed in the baseline characteristics of patients included (surroundings, race, age, sex and treatment). Thus a random effects model analysis was employed to compensate for these deficiencies.

In conclusion, our meta-analysis is the first study to systematically estimate the association between p53 positivity and survival of SACC. As determined in our meta-analysis, it was concluded that p53 expression was associated with histological subtypes, tumor stage, metastasis, local recurrence, nerve infiltration and 5-year OS in SACC. Tumor of the solid subtype had a strong positive correlation with p53 expression. Besides, compared to patients with p53-negative expression, those with p53-positive expression had more chance of developing metastasis, local recurrence, nerve infiltration and poorer 5-year OS. To strengthen our findings, well-designed prospective studies with better standardized assessment of prognostic markers are required to explore the relationship between p53 expression and survival of SACC. Hence, the above mentioned parameters can be considered important while planning the management which may need an aggressive approach in these cases. More researches are required for additional comprehensive prognostic assessment of SACC.

## MATERIALS AND METHODS

### Search strategy and study selection

The electronic databases PubMed, Embase, Cochrane Library, Web of Science and China National Knowledge Infrastructure were searched for studies to be included in the present meta-analysis. An upper date limit of June 2016 was applied, and no lower date limit was set. The terms of “ACC or Adenoid Cystic Carcinoma”, “salivary glands or sialaden”, “cancer or carcinoma or tumor or neoplasm”, “p53” were used for searching. Relevant articles, abstracts, and review articles were selected and reviewed, and the reference lists from these sources were searched for additional trials. We also reviewed the Cochrane Library for relevant articles. The references listed in the identified studies were also used to complete the search.

All candidate studies were reviewed by two independent reviewers (Li QL and Wang JF). Discrepancies were resolved by discussion. Our search was initially narrowed based on the title followed by the abstract, and finally full papers were reviewed if they were categorized as relevant studies. All of the references from review papers and original reports were examined for further relevant studies. Studies eligible for inclusion in this meta-analysis met the following criteria:(1): The object of literature study should be human salivary gland tumor, and adenoid cystic carcinoma must be contained;(2) Immunohistochemistry (IHC) was used to measure p53 expression in the tumor tissue;(3) there was a standard to judge if p53 expression was positive or negative in the tumor tissue;(4) the full paper can be obtained;(5)when the same author reported the same patient population in more than one publication, only the most recent report or the most complete one was included in the analysis. Exclusion criteria: (1) the relationship between ACC and P53 expression was not investigated; (2) repetitively published literature; (3) the study in which the corresponding data cannot be extracted from the original literature; (4) animal experiment or a single case report.

### Data extraction and quality assessment

The final articles included were assessed independently by two reviewers (Li QL and Wang JF). Disagreements were addressed by discussion with a third reviewer until the first two reviewers reached a consensus or by contacting experts if necessary. Data retrieved from the reports included the first author's name, publication year, patient source, control source, detection method, number of samples and histopathological patterns. If data from any of the above categories were not reported in the primary study, items were treated as “not applicable.”We did not contact the author of the primary study to request the information.

Quality assessment of included primary studies was independently performed by two reviewers (Li QL and Wang JF) using the Newcastle-Ottawa Quality Assessment Scale (NOS) [54]. NOS scores of ≥ 6 were defined as high-quality studies. Any disagreement was solved by discussion.

### Statistical analysis

Review manager software (Version 5.2, The Cochrane Collaboration, Copenhagen, Denmark). and the Stata 12.0 statistical software (Stata Corporation, College Station, TX, USA) were used to perform the meta-analysis. Cochrane's Q statistic was used to evaluate the heterogeneity of the primary studies. The main results were displayed in the forest plots. If heterogeneity was not considered to be statistically significant (p>0.10 or I^2^<50%), the data were analyzed using a fixed-effect model; otherwise, a random-effect model was chosen. Publication bias was first assessed by visual judgment of a funnel plot, and Begg's test was then performed for each pooled study groups. A series of sensitivity analyses were performed to determine the impact of pooled models or trials with incompatible factors on the overall results. The OR was calculated for all of the analyses. A statistical test with a p-value less than 0.05 was considered significant.
